# FcRL4 Expression Identifies a Pro-inflammatory B Cell Subset in Viremic HIV-Infected Subjects

**DOI:** 10.3389/fimmu.2017.01339

**Published:** 2017-10-20

**Authors:** Basile Siewe, Allison J. Nipper, Haewon Sohn, Jack T. Stapleton, Alan Landay

**Affiliations:** ^1^Department of Immunology and Microbiology, Rush University Medical Center, Chicago, IL, United States; ^2^Laboratory of Immunogenetics, National Institute of Allergy and Infectious Diseases, National Institutes of Health, Rockville, MD, United States; ^3^Iowa City Veterans Affairs Medical Center, Department of Internal Medicine, University of Iowa, Iowa City, IA, United States; ^4^Iowa City Veterans Affairs Medical Center, Department of Microbiology and Immunology, University of Iowa, Iowa City, IA, United States

**Keywords:** Fc receptor-like 4, pro-inflammatory cytokine, HIV, B cells, IL-6, viremic

## Abstract

In autoimmune diseases, toll-like receptor (TLR)-stimulated pro-inflammatory IL-6-secreting B cells exert pathogenic roles. Similarly, B cell Fc receptor-like 4 (FcRL4) expression amplifies TLR stimulation, and in rheumatoid arthritis patients, FcRL4 expression identifies a pro-inflammatory B cell subset. B cells from HIV-infected subjects also express heightened levels of FcRL4 and secrete high levels of IL-6: a critical mediator of HIV disease progression. In this study, we sought to determine if FcRL4 identifies a pro-inflammatory B cell subset in HIV-infected subjects and further elucidate the mechanisms underlying FcRL4 amplification of TLR stimulation. We determine that tissue-like memory B cells express the highest endogenous levels of FcRL4 positively correlating with IL-6 expression (*p* = 0.0022, *r* = 0.8667), but activated memory B cells exhibit the highest frequency of FcRL4^hi^IL-6^hi^ cells. FcRL4^hi^ B cells exhibit an activated TLR-signaling pathway identified by elevated phosphorylation levels of: pERK (*p* = 0.0373), p38 (*p* = 0.0337), p65 (*p* = 0.1097), and cJUN (*p* = 0.0239), concomitant with significantly elevated expression of the TLR-signaling modulator hematopoietic cell kinase (HcK, *p* = 0.0414). Compared to FcRL4^neg^ B cells from healthy controls, TLR9-stimulated FcRL4^pos^ B cells express significantly higher levels of lL-6 (*p* = 0.0179). Further, TLR9-stimulated B cells also upregulate the expression of FcRL4 (*p* = 0.0415) and HcK (*p* = 0.0386). In B-cell lines, siRNA-mediated HcK knockdown downmodulates TLR9-induced FcRL4-mediated activation quantified by CD23 upregulation (*p* = 0.0553). We present data suggesting that, in viremic HIV-infected individuals, FcRL4 expression identifies unique IL-6 producing pro-inflammatory B-cell subsets. Further, TLR stimulation likely modulates FcRL4 expression and FcRL4 expression is associated with Hck, potentially enhancing the activation of TLR-signaling associated transcription factors. Pathogenic B-cells have been identified in other disease settings, and this study represents a novel report describing a pro-inflammatory B cell subset in HIV-infected patients.

## Introduction

The elevated serum level of the pro-inflammatory cytokine IL-6 is an indicator of chronic immune activation and a driver of HIV disease progression (1, 2). During HIV infection, IL-6 overexpression drives B-cell proliferation, enhances secretion of antibodies, and leads to aberrant B cell terminal differentiation (3, 4). Further, *in vitro*, IL-6 has been shown to drive HIV replication and, in HIV-infected individuals, the observed high levels of IL-6 are associated with increased mortality and morbidity (5, 6). Due to these factors, it is critical to determine the sources of IL-6 as well as the mechanisms underlying IL-6 overexpression during HIV infection. HIV infection is characterized by heightened microbial translocation and the presence of microbial products encoding toll-like receptor ligands (TLR-L) (7–9). TLR-stimulated monocytes have been identified to be a significant contributor to the HIV-induced inflammatory state (10–12). However, published data also suggest that B cells from HIV-infected individuals express high levels of IL-6 possibly due to TLR-stimulation (3, 9, 13). Additionally, in autoimmune diseases, TLR-stimulated B-cells are critical mediators of inflammation (14, 15). Further, data from a study in rheumatoid arthritis identified a pro-inflammatory B-cell subset expressing high levels of Fc receptor-like 4 (FcRL4) (16). FcRL4 acts as a molecular switch, dampening B cell receptor (BCR) signaling while simultaneously enhancing TLR-signaling through association of SHP-1 and SHP-2 with its cytoplasmic tail (17). Finally, B cells from HIV-infected viremic subjects exhibit heightened FcRL4 expression associated with an “exhausted” phenotype, with impaired antibody expressing functions (18–20).

In this study, we investigated: (1) if in untreated HIV infection, FcRL4^hi^ B-cells represent a pro-inflammatory B cell subset and (2) the mechanisms underlying FcRL4 expression and amplification of TLR-signaling. Our data indicate that FcRL4^hi^ B-cell subsets are high producers of IL-6, and TLR-signaling modulates FcRL4 expression. Finally, FcRL4 mediates amplification of TLR-signaling likely by recruiting Src Kinase proteins.

## Materials and Methods

### Study Participants

All studies were performed after signed, informed written research consent by each study subject. The study was reviewed and approved by the Institutional Review Board of the Rush University Medical Center, and the University of Iowa City VAMC and University of Iowa. All work was performed in adherence with appropriate laboratory safety protocols such as use of personal protective equipment. HIV-infected viremic (HIV_VIR_), naïve subjects had a median CD4 count of 466 cells/μl (range, 144–566), and median viral load of 20,000 copies/ml (range, 2,000–117,000) (Table 1).

**Table 1:** HIV viremic cohort description.

Participant	Age (years)	Gender	CD4 count (cells/μL)	Viral load (copies/mL)
1	40	M	566	10,000
2	27	F	443	80,000
3	45	F	515	117,000
4	57	M	592	14,000
5	46	M	337	38,000
6	39	M	550	3,000
7	46	F	217	34,000
8	36	M	144	2,000
9	23	M	489	5,000
10	56	F	288	26,000

*M, male; F, female*.

### Cell Lines

Ramos (a human Burkitt lymphoma cell line) FcRL4 stable transfectants were a generous gift from Dr. Susan Pierce (NIH) and previously described (17). The FcRL4.FFF mutant carries mutations (tyrosine to phenylalanine) in the cytoplasmic ITIM tail at positions 451, 463, and 493. The cells were maintained in RPMI medium supplemented with 10% FBS, Pen/Strep, 2mM l-glutamine, 10 mM HEPES, and 55 µM β-mercaptoethanol (Invitrogen).

### Antibodies

Cells were stained with the following antibodies: FcRL4-APC (Biolegend), IL-6-PE, CD23-PE-Cy7, CD19-PE-Texas Red, CD10-Pe-Cy5, CD21-V450, CD27-AF700, phospho-p38-PE, phospho-Erk-AF647, phospho-p65-PE, phospho-C-Jun-FITC, (BD Biosciences), Sheep anti-rabbit IgG-DyLight 488 (Biolegend), purified hematopoietic cell kinase (Hck), and phospho-Hck (Abcam).

### Isolation, Purification, and TLR Stimulation of PBMCs

PBMCs were isolated from whole blood using Ficoll (Lymphocyte^®^ Cell Separation Media, Mediatech) gradient centrifugation. Cryopreserved PBMC from HIV-infected subjects were used in the immunophenotyping experiments. The cells were stained with FcRL4-APC and CD19-PE-Texas Red and CD19^+^FcRL4^pos^ and CD19^+^FcRL4^neg^ B cells were FACS purified and cultured overnight in the presence of 10 µg/ml CpG-B ODN2006 (TLR9L), 2 µg/ml PAM3CSK4 (TLR2L), or 2 µg/ml Imiquimod (InvivoGen). B cells (CD19^+^) from healthy controls were purified from PBMC using the B Cell Isolation Kit II (Miltenyi Biotec) and AUTOmacs (Miltenyi Biotec). After 4H, the cultures of CD19^+^ B cells were supplemented with Brefeldin A (1:1,000, BD). After overnight incubation, the cells were surface stained (CD23-PE-Cy7, BD Biosciences), fixed/permeabilized (Fix/Perm Kit BD Biosciences), and stained for intracellular IL-6 (IL-6-PE, BD Biosciences). All samples were acquired on an LRSII (BD Biosciences) flow cytometer and the data analyzed using FlowJo software (Tree Star Inc.). Florescence parameters were normalized using Rainbow Calibration Particles (Spherotech) and antibody bound CompBead (BD Biosciences). Gating was determined by unstained controls.

### Inhibition Assays

Chemical inhibition of Hck was achieved using PP2 (Millipore). Cells were incubated overnight with indicated concentrations of the inhibitor, supplemented with TLR9-L, and further cultured overnight. Only events corresponding to living cells (determined by Live/Dead^®^ Fixable Aqua staining, Life Technologies) were acquired on an LRSII (BD Biosciences) flow cytometer and the data analyzed using FlowJo software (Tree Star Inc.).

### Real-time RT-PCR

RNA was extracted using the RNeasy Kit (QIAGEN) according to the manufacturer’s instructions. The extracted RNA was measured by spectrophotometer and equimolar concentrations used for cDNA synthesis according to the manufacturer’s instructions (iScript cDNA syntesis Kit, Bio-Rad). The following primers were used for the qPCR reaction: HcK-Forward 5′-CGGATCCCACATCCACCATCA-3′, Reverse 5′-ACCACGATGATGTCCTCAGAGC-3′, FcRL4-Forward 5′-TCAGCTGGGAGAAGAAGAGGAA-3′, Reverse 5′-GAGTTATCTGGGTGTTGTGTCTTTACC-3′, GAPDH-Forward 5′-CTTCAACGACCACTTTGT-3′ and reverse 5′-TGGTCCAGGGGTCTTACT-3′. Real-time RT-PCR was performed using a Quantitect SYBR Green PCR kit (Qiagen) in a 7900HT Fast Real-Time PCR system (Applied Biosystems). Melting curve analysis was performed to ensure that the primers amplified the desired amplicon and that primer-dimers were absent. Fold change in mRNA expression was calculated by relative quantification using the comparative cycle threshold method. *GAPDH* expression was used as an endogenous control.

### siRNA-Mediated Knockdown

siRNA targeting HcK were purchased from Santa Cruz Biotechnology and Dharmacon, and cells were transfected using the Lipofectamine RNAiMax kit (Life Technologies) according to the manufacturer’s instructions. Knockdown was confirmed by qPCR 48H post-transfection.

### Statistical Analysis

Results are expressed as mean ± SEM or as indicated. GraphPad Prism software, version 5.03 was used for all statistical analysis. The statistical significance *p-*value between group parameters was determined using either unpaired or paired Student’s *t-*test (with a confidence level of 95%). The statistical dependence between variables was calculated using the Spearman rank correlation analysis. *p-*Values of <0.05 were considered statistically significant. Pair and multiple comparisons were done using the Wilcoxon-matched-pairs signed rank test.

## Results

### FcRL4^hi^ B-Cell Subsets from HIV-Infected Viremic Subjects Spontaneously Express High Levels of IL-6

**Figure 1:**
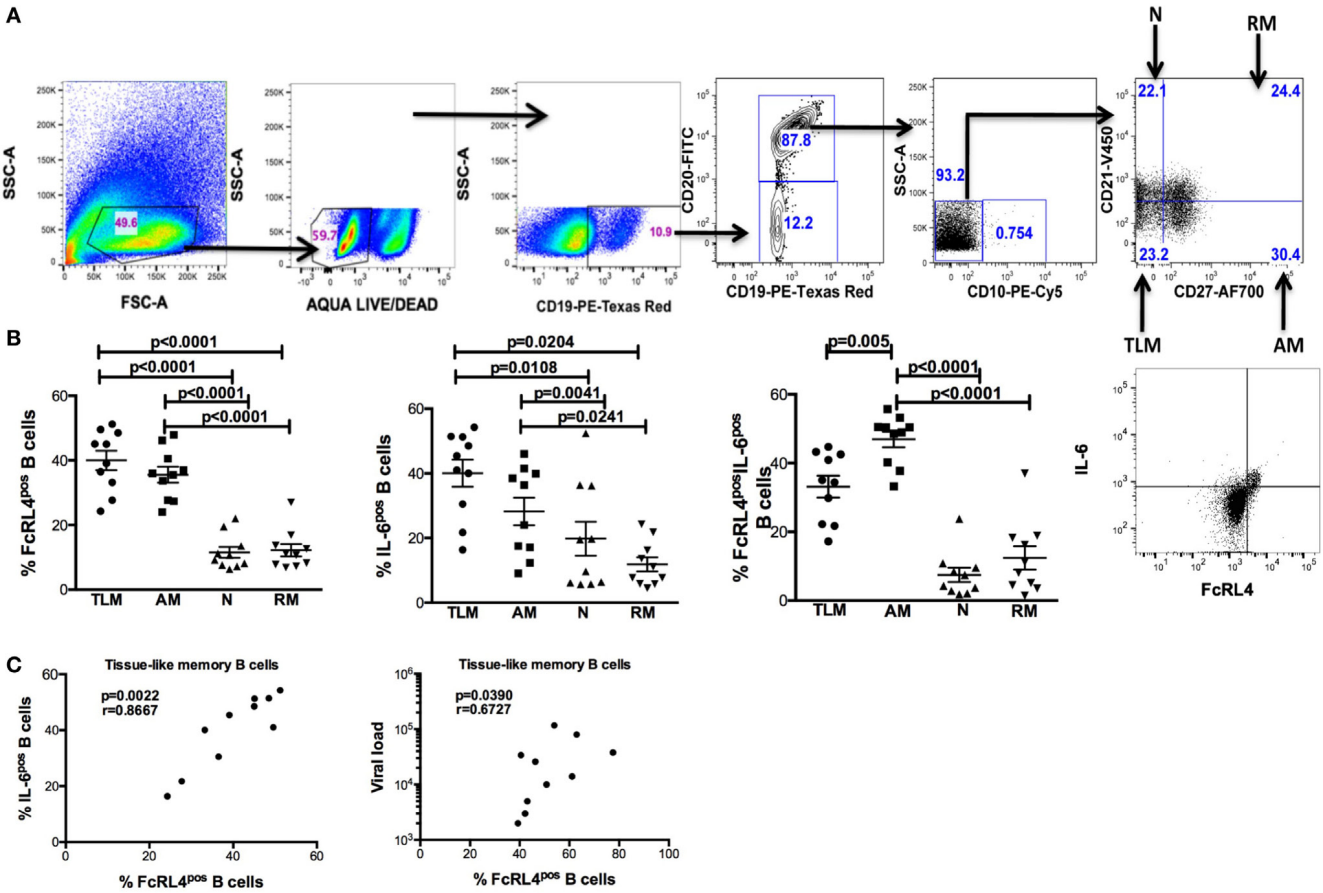
FcRL4^hi^ blood B cell subsets from HIV viremic subjects express high endogenous levels of IL-6. In blood B cell subsets [**(A)**, gating strategy] from HIV viremic subjects (*n* = 10), the endogenous levels of **(C)** FcRL4, IL-6, and FcRL4^+^IL-6^+^ [representative plots depicted in **(B)**] as well as **(C)** the relationship between FcRL4 expression and IL-6 (left) and viral load (right) on tissue-like memory B cells were determined; N, naïve; RM, resting memory; AM, activated memory; TLM, tissue-like memory. *p-*Values as determined by Mann–Whitney test are indicated, in **(C)** association was calculated using the spearman correlation.

In rheumatoid arthritis patients, FcRL4 expression identifies a pro-inflammatory B-cell subset (16). Differential FcRL4 expression among B cell subsets has been reported in HIV viremic (HIV_VIR_) subjects (18); however, the relationship between FcRL4 expression and production of pro-inflammatory cytokines has not been fully elucidated. Our prior data indicate that B cells from HIV-infected individuals express primarily IL-6 and not TNF-α (9). We investigated if FcRL4 expression on B cell subsets from HIV_VIR_ subjects associated with heightened endogenous levels of IL-6 expression. Tissue-like memory B cells (TLM, CD19^+^CD20^+^CD10^−^CD21^lo^CD27^−^) expressed the highest levels of FcRL4 among different B cell subsets (Figures 1A,B), comparable to activated memory B cells (AM, CD19^+^CD20^+^CD10^−^CD21^−^CD27^+^), but significantly higher than naïve B cells (N, CD19^+^CD20^+^CD10^−^CD27^−^CD21^+^, *p* < 0.0001) and resting memory B cells (RM, CD19^+^CD20^+^CD10^-^CD21^+^CD27^+^, *p* < 0.0001). TLM B cells also expressed the highest endogenous levels of IL-6 (Figure 1B) compared to naïve (*p* = 0.01081) and RM B cells (*p* = 0.0204). Likewise, in AM B cells (Figure 1B); the level of IL-6 was much higher as compared to naïve (*p* = 0.0041) and RM B cells (*p* = 0.0241). Moreover, AM cells expressed the highest frequency of FcRL4^+^IL-6^+^ cells (Figure 1B): significantly higher than TLM (*p* = 0.005), N (*p* < 0.0001) and RM (*p* < 0.0001) B cells. Taken together, TLM and AM B cells express the highest levels of FcRL4 and IL-6 as well as the frequency of FcRL4^+^IL-6^+^ cells. Finally, in the TLM B cells, we observed a significant positive correlation between the FcRL4 and IL-6 expression (Figure 1C, *p* = 0.0022, *r* = 0.8667) as well as FcRL4 and HIV viral load (Figure 1C, *p* = 0.0390, *r* = 0.6727).

### FcRL4^pos^ B Cells from HIV-Infected Viremic (HIV_VIR_) Subjects Constitutively Exhibit an Activated TLR-Signaling Cascade

**Figure 2:**
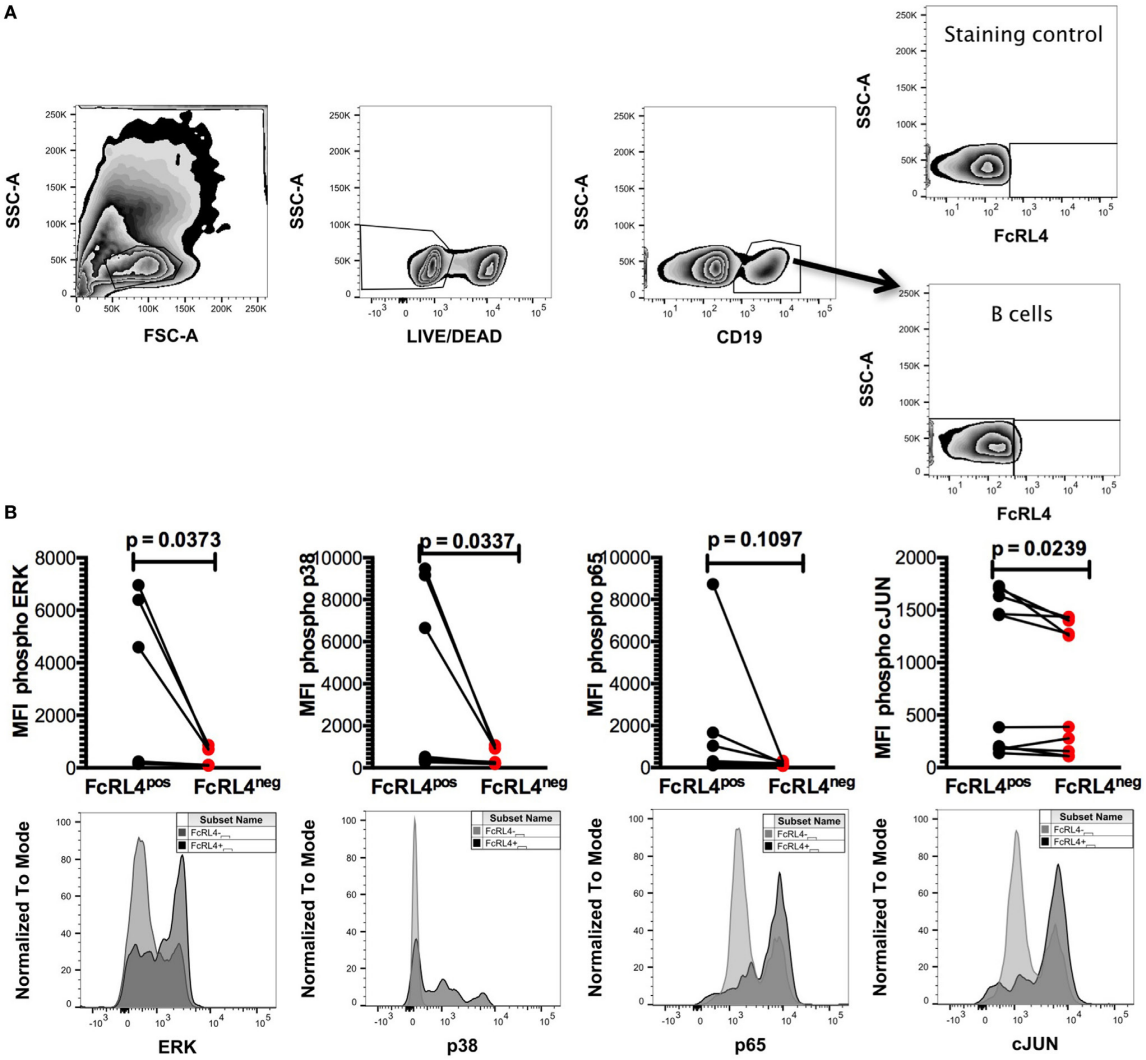
FcRL4^pos^ B cells from HIV viremic subjects are Hck^hi^ and exhibit activated TLR-signaling signature. In FcRL4^pos^, B cells from HIV viremic subjects (*n* = 10) **(A)** gating strategy for FcRL4, using flow cytometry, **(B)** the endogenous activation levels of MAPK pathway members Erk and p38 as well as activation of NF-κB (p65) and AP-1 (c-Jun) were determined: Representative overlays of FcRL4^-^ and FcRL4^+^ populations shown. *p-*Values as determined by Mann–Whitney test are indicated.

HIV-infection is associated with an increase in serum concentration of several TLR ligands (7–9), and B cells from HIV_VIR_ individuals exhibit enhanced FcRL4 expression (18). As FcRL4 enhances B-cell responsiveness to TLR stimulation (17), we next investigated if, in HIV_VIR_ subjects, constitutive FcRL4 expression is associated with an activated TLR-signaling pathway. We determined that FcRL4^pos^ B cells of HIV_VIR_ subjects exhibit a constitutively activated TLR-signaling pathway phenotype characterized by significantly elevated levels of phosphorylated ERK, p38, and c-JUN (Figures 2A,B, *p* = 0.0373, *p* = 0.0337, and *p* = 0.0239, respectively). Although the level of phosphorylated p65 was higher in FcRL4^pos^ B cells than FcRL4^neg^ B cells, the difference did not attain statistical significance (Figure 2B, *p* = 0.1097).

### FcRL4^pos^ B Cells from HIV-Uninfected Subjects Are Highly Responsive to TLR Stimulation

**Figure 3:**
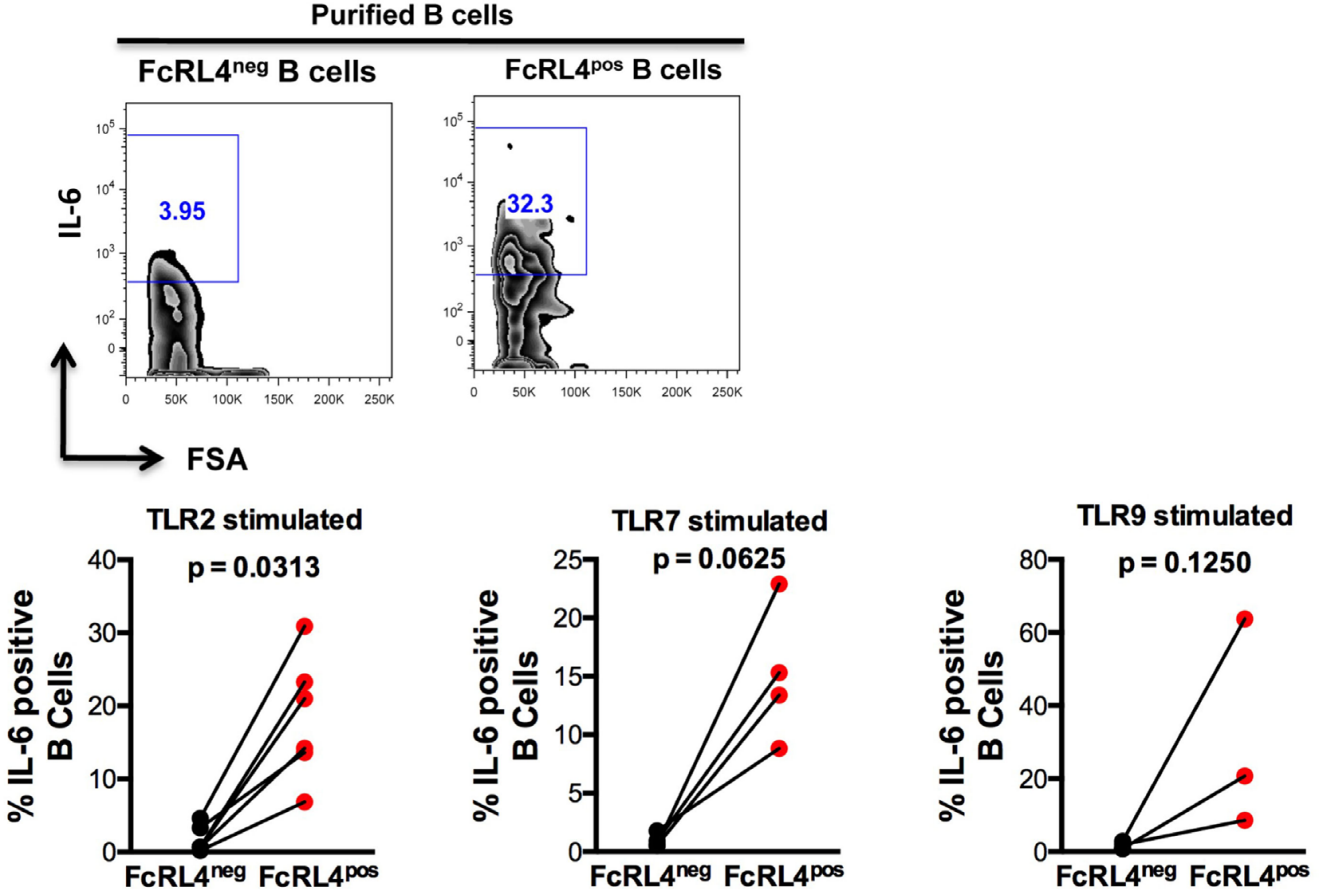
FcRL4^pos^ B cells from HIV-uninfected subjects are highly responsive to TLR stimulation. FcRL4^hi^ and FcRL4^lo^ B cells were enriched from primary B cells of HIV_neg_ subjects, exposed to TLR2-Ligand (TLR-2 stimulated, 2 µg/ml, Pam_3_Csk_4_), TLR7L (TLR-7 stimulated 2 µg/ml, Imiquimod), or TLR9-ligand (TLR-9 stimulated 10 µg/ml CpGODN2006). After overnight incubation, IL-6 expression was determined by intracellular cytokine staining. Representative plots are shown in the top panels, in lower panels, each dot represents a subject (*n* = 6, 4, 3 from left to right), *p-*values as determined by paired *t*-test are indicated.

We previously demonstrated that TLR stimulated B cells from healthy controls (HIV_NEG_) subjects upregulate expression of the pro-inflammatory cytokine IL-6 (9). We, therefore, examined if FcRL4 modulates the expression of IL-6 upon TLR stimulation. We found that compared to FcRL4-negative (FcRL4^neg^) B cells, TLR stimulation of purified FcRL4^pos^ B cells significantly upregulated IL-6 expression (Figure 3: TLR2, *p* = 0.0022, TLR7, *p* = 0.0286, TLR9, *p* = 0.0179).

### B Cells Exposed to TLR-9 Ligand Upregulate Expression of FcRL4 and HcK Concomitantly

**Figure 4:**
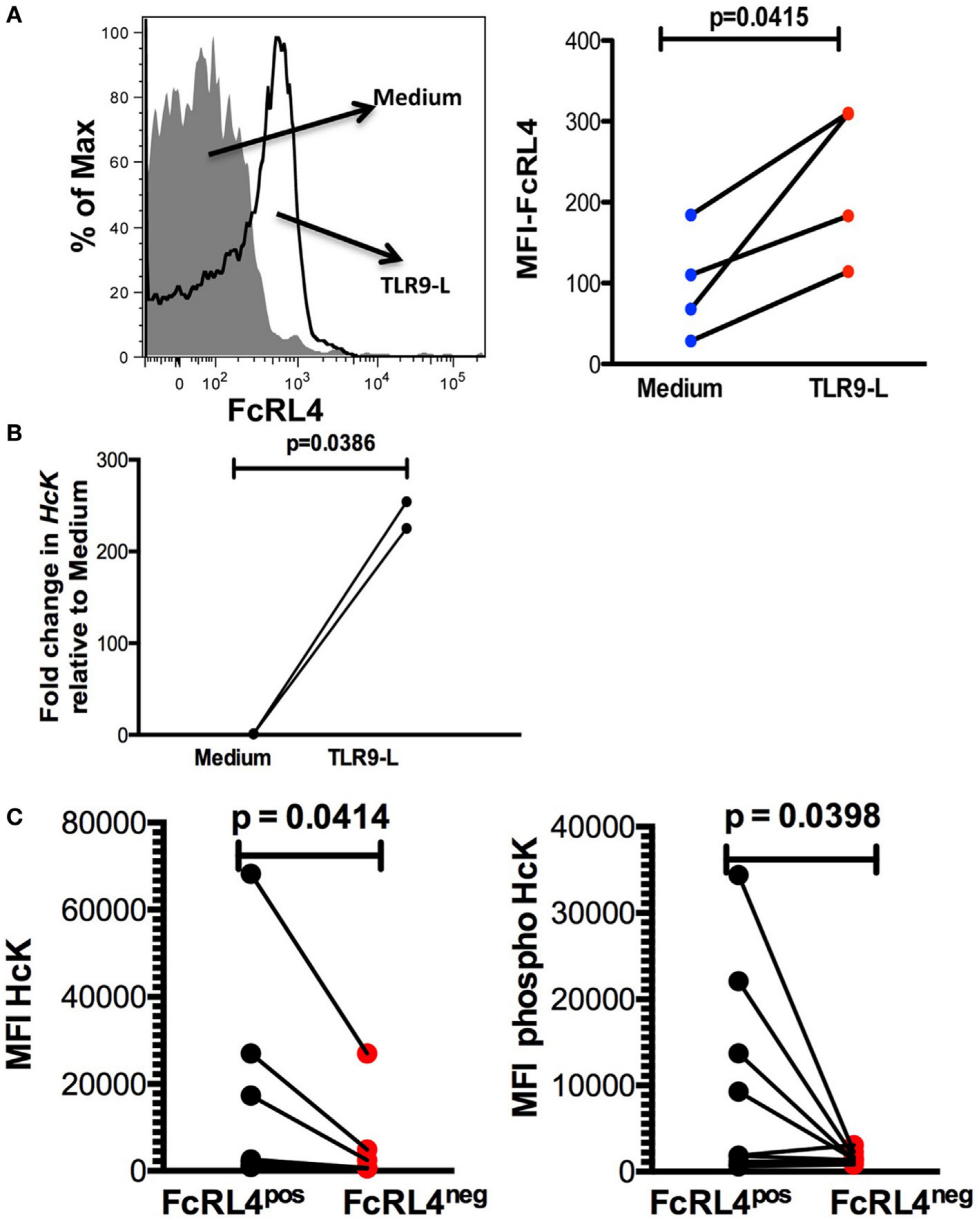
TLR-stimulated B cells upregulate FcRL4 and hematopoietic cell kinase (Hck) expression. Purified B cells from HIV_neg_ subjects were stimulated overnight with 10 µg/ml CpGODN 2006 and the expression of **(A)** FcRL4 and **(B)** *Hck* determined by flow-cytometry and RT-qPCR, respectively. Each dot represents a subject. **(C)** In FcRL4^pos^, B cells from HIV viremic subjects (*n* = 10, Figure 2A, gating strategy for FcRL4), the endogenous level of total HcK (left) and phosphorylated HcK (right) expression were determined. *p-*Values as determined by paired *t*-test are indicated.

Elevated FcRL4 expression on blood B cells has been identified in malaria and HIV-infected viremic patients (18, 19), conditions associated with heightened serum levels of TLR ligands (7, 8, 21). Additionally, it has been previously demonstrated that TLR stimulation modulates FcRL expression in mice (22). We determined that exposure of PBMC from HIV_NEG_ subjects to TLR9 stimulation led to a significant upregulation in FcRL4 expression (Figure 4A, *p* = 0.0415). We confirmed that while TLR stimulation induces FcRL4 upregulation, the anti-FcRL4 flow cytometry antibody did not lead to FcRL4 upregulation. In human secondary lymphoid tissue, elevated FcRL4 expression is associated with heightened levels of the Src kinase family member HcK (23), which in macrophages, promotes TLR-induced expression of pro-inflammatory cytokines (24). We, therefore, investigated if in TLR-stimulated blood B cells, the observed FcRL4 upregulation (Figure 4A) is associated with heightened HcK expression contributing to the amplification of the TLR-signaling. We determined that TLR9-stimulation of purified B cells from HIV_NEG_ resulted in the upregulation of *HcK* levels (Figure 4B, *p* = 0.0386) (gating Figure S2 in Supplementary Material). Finally, in HIV_VIR_ subjects, FcRL4^pos^ B cells, expressed significantly higher endogenous levels of total (Figure 4C, *p* = 0.0414) and phosphorylated HcK (Figure 4C, *p* = 0.0398).

### HcK Is Required for FcRL4-Mediated Amplification of TLR Signaling

**Figure 5:**
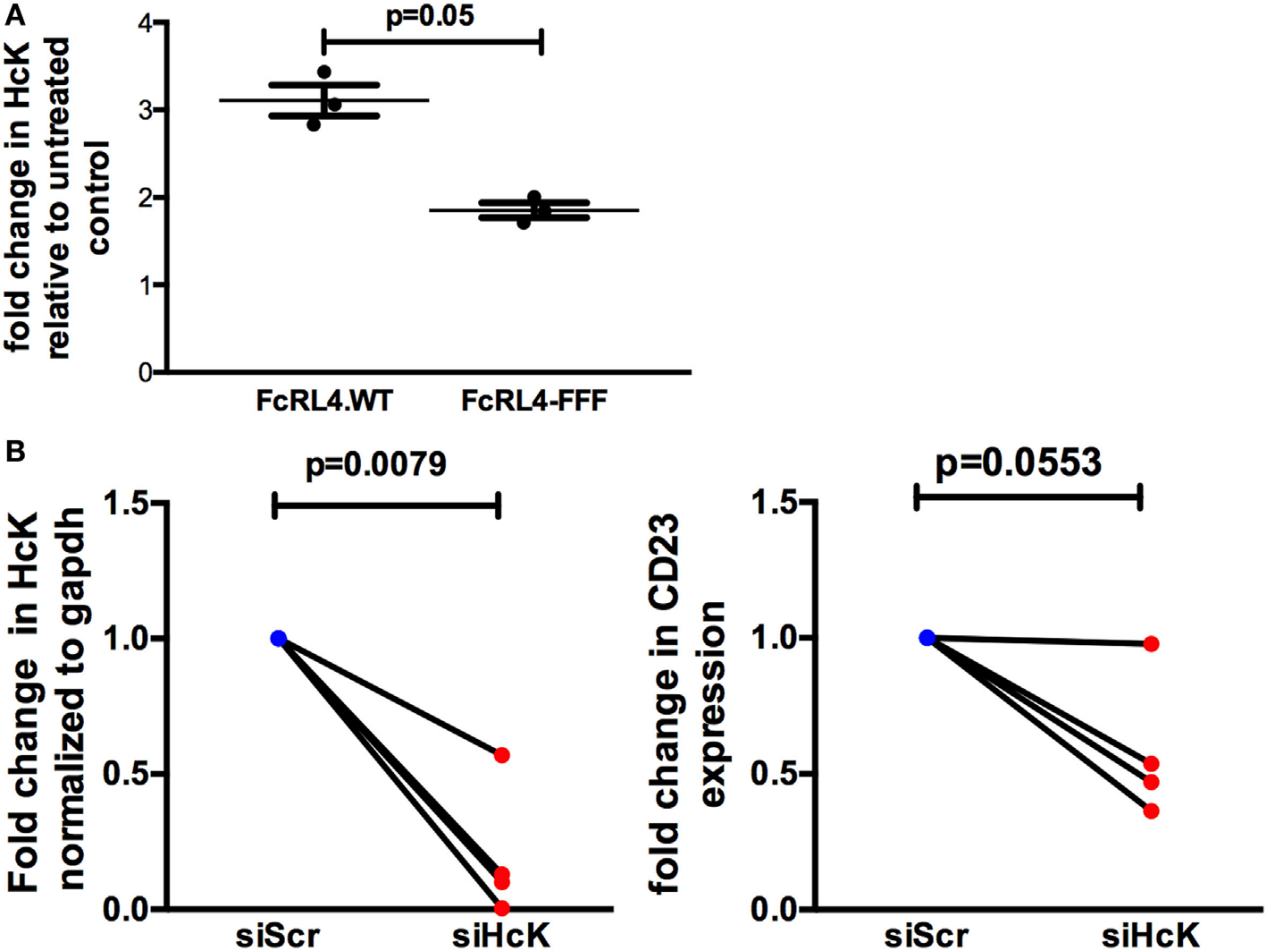
Hematopoietic cell kinase (HcK) is necessary for FcLR4-mediated amplification of TLR signaling. In FcRL4 transfectants, **(A)** HcK expression was determined after TLR9 stimulation by flow cytometry, *n* = 3. **(B)** *Hck* expression was determined by RT-qPCR post RNAi (left); siHCK and control RNAi treated cells were incubated overnight with TLR9-L and CD23 expression detected by flow-cytometry (right) shown as fold change. siScr = scrambled control; *n* = 4. *p*-Values as determined by paired *t*-test are indicated.

The effect of HcK on TLR-signaling in B cells was further investigated using a B cell line stably expressing FcRL4 (FcRL4.WT) and a loss-of-function FcRL4 mutant cell line, incapable of amplifying TLR-signaling (FcRL4.FFF) (17). We determined that after TLR stimulation, HcK upregulation was evident only in the FcRL4.WT cells (Figure 5A). *HcK* expression in FcRL4.WT transfectants was reduced using siRNA and confirmed by qPCR (Figure 5B, *p* = 0.0079, compared to control). Finally, TLR9 activation was quantified by change in CD23 expression, a readout of TLR9 activity (17). HcK knockdown led to a reduction in CD23 expression (Figure 5B, *p* = 0.0553) following TLR9 stimulation. To confirm these results, we chemically inhibited HcK using 4-amino-5-(4-chlorophenyl)-7-(t-butyl)pyrazolo[3,4-d]pyrimidine (PP2) as described elsewhere (25, 26). HcK chemical inhibition reduced TLR9-induced CD23 expression significantly in FcRL4.WT compared to FcRL4.FFF in a dose-dependent manner (Figure S1 in Supplementary Material, 1 mM and 10 mM PP2, *p* = 0.0059 and *p* = 0.0052, respectively).

## Discussion

In this study, we demonstrate that during viremic HIV infection, FcRL4^hi^ TLM and AM blood B cells express high endogenous levels of IL-6, strongly indicating that high FcRL4 expression identifies pro-inflammatory B cells. We demonstrate that frequency of FcRL4^+^ B-cells correlates strongly with IL-6^+^ B-cell frequency in the TLM subset. However, AM B cells exhibit the highest frequency of FcRL4^+^IL-6^+^ double-positive cells suggesting the possibility that divergent mechanisms drive IL-6 production in AM and TLM B cells. This concept of divergent mechanisms is further supported by the distinct characteristics of these subsets, with TLM displaying elevated expression of inhibitory receptors and increased frequency of HIV-specific B cells, while the AM subset show greater specificity for other pathogens (20). Taken together, our report identifies pro-inflammatory functions of FcRL4^+^ TLM B cells in viremic HIV-infected subjects, corroborating findings, which identify FcRL4^hi^ B cells as a marker of pro-inflammatory B cells in rheumatoid arthritis patients (16).

Though FcRL4 has previously been identified on exhausted B-cell subsets (20), weak proliferation following BCR stimulation may be indicative of a shift in function rather than a general failure to respond. FcRL4 has been identified as a molecular switch, dampening BCR signaling while enhancing B-cell responsiveness to TLR-stimulation (17). HIV-infected viremic (HIV_VIR_) subjects exhibit elevated serum levels of TLR-ligands (7–9) concomitant with high expression of FcRL4 on B cells (18, 20). It is, therefore, tempting to suggest that in HIV_VIR_ subjects, TLM and AM B cells are stimulated by TLR-ligands resulting in upregulated FcRL4 expression. This increases sensitivity to TLR stimulation, leading to a positive feedback loop culminating in high expression of IL-6, inflammation, and HIV disease progression. Though we cannot exclude the possibility that FcRL4-expressing B cells coincidently express IL-6, our data provide further evidence supporting a role for FcRL4 in mediating *in vivo* TLR-signaling-dependent hyperstimulation during HIV infection. We also determined that *ex vivo*, FcRL4^hi^ B cells from HIV_VIR_ subjects exhibit a TLR-signaling signature, characterized by heightened activation of NF-κB and AP1 pathways, transcription factors critical for the expression of pro-inflammatory genes (27–29).

While FcRL4 expression has been well documented in HIV, its function remains only partly elucidated. During HIV-1 infection, FcRL4 is elevated on TLM of non-treated individuals, but expression is greatly reduced following treatment (30); this suggests a unique role for FcRL4 during HIV infection. Jelicic et al. report that HIV gp120 induces FcRL4 expression on B cells (31), suggesting another mechanism inducing FcRL4 expression, which enhances susceptibility to TLR stimulation in HIV infection. Previous studies also suggest that another FcRL family protein, FcRL3, is upregulated in response to TLR stimulation (32); however, a role for TLR stimulation in regulating FcRL4 expression in HIV infection has not been explored. We provide data suggesting that TLR-signaling augments B-cell FcRL4 expression, corroborating reports of TLR-regulation of FcRL3 (32). Though we present data indicating B cells exposed to TLR9-ligand CpG-ODN2006 upregulate FcRL4 expression, we also observed comparable effects when B cells are exposed to either TLR7 (Imiquimod) or TLR2 (Pam_3_Csk_4_) ligands (not shown). Sohn et al. elegantly demonstrated that FcRL4 expression switches B-cell responsiveness from adaptive to innate stimulus (17); however, the underlying mechanism is still undefined. Our data present a potential mechanism underlying FcRL4-mediated amplification of TLR-signaling in B cells. Ehrhardt et al. (23) reported that human tissue FcRL4^hi^ B cells concurrently express high levels of the Src-kinase family member HcK, and Smolinska et al. (24) determined that Hck recruitment amplifies TLR4 signaling in macrophages. Our data confirm these findings, as we show that TLR9-stimulated B cells from HIV_NEG_ subjects upregulate HcK and FcRL4^hi^ B cells from HIV_VIR_ subjects exhibit elevated endogenous levels of HcK. Further, HcK downmodulation resulted in a reduction of TLR-signaling in FcRL4 B-cell transfectants. These data confirm recent reports by Liu et al. (33) and suggest that FcRL4 in human B cells likely recruits the Src-kinase family member HcK, resulting in amplification of TLR-signaling. However, further studies are needed to determine the precise association between FcRL4 and HcK. Our finding that TLR9-stimulation was impervious to HcK chemical inhibition in the FcRL4.FFF loss-of-function mutant suggests a role for the ITIM in HcK recruitment following TLR-signaling, as FcRL4 of this mutant is incapable of specific ITIM phosphorylation events.

B cells have been well established as a critical source of pro-inflammatory IL-6 in autoimmune diseases (14), and some reports also suggest that during HIV infection B-cells express IL-6, thus likely exerting a pathogenic role (3, 9, 13). Our data present FcRL4 as a marker identifying potential pro-inflammatory B cells during viremic HIV infection.

## Conclusion

The data from this study indicate that in viremic HIV infected subjects, high expression of FcRL4 identifies pro-inflammatory B cell subsets. In autoimmune conditions, B cells have been established as critical IL-6 expressing cells (16). Our data demonstrate a pro-inflammatory function of FcRL4^+^ B cells, a population of B cells previously identified as exhausted, in viremic HIV infection. Finally, we present data elucidating the mechanisms of FcRL4-mediated amplification of TLR-signaling in B cells. We provide data indicating that increased expression of FcRL4 coincides with upregulation of the Src kinase HcK, and HcK is necessary for FcRL4’s amplification of TLR signaling.

## Ethics Statement

All studies were performed after signed informed written research consent by each study subject. The study was reviewed and approved by the Institutional Review Board of the Rush University Medical Center, and the University of Iowa City VAMC and University of Iowa.

## Author Contributions

BS and AL conceived/designed study and wrote manuscript. BS and AN performed experiments. BS, AN, AL, HS, and JS analyzed data and edited manuscript.

## Conflict of Interest Statement

The authors declare that the research was conducted in the absence of any commercial or financial relationships that could be construed as a potential conflict of interest. The reviewer CS and handling editor declared their shared affiliation.

## Supplementary Material

Figure S1 CD23 expression following overnight treatment with HcK inhibitor PP2. FcRL4.WT and FcRL4.FFF cells were treated with TLR9-L and HcK inhibitor. Expression of CD23 reported as fold change between TLR9-L alone and in the presence of inhibitor *n*=3.

Figure S2 Representative gating of HcK and phospho HcK.

## References

[1] Leeansyah E Malone DF Anthony DD Sandberg JK. Soluble biomarkers of HIV transmission, disease progression and comorbidities. Curr Opin HIV AIDS (2013) 8:117–24. 10.1097/COH.0b013e32835c7134 23274365

[2] Langford SE Ananworanich J Cooper DA. Predictors of disease progression in HIV infection: a review. AIDS Res Ther (2007) 4:11. 10.1186/1742-6405-4-11 17502001 PMC1887539

[3] Rieckmann P D’Alessandro F Nordan RP Fauci AS Kehrl JH. IL-6 and tumor necrosis factor-alpha. Autocrine and paracrine cytokines involved in B cell function. J Immunol (1991) 146:3462–8. 2026875

[4] Heinrich PC Castell JV Andus T Interleukin-6 and the acute phase response. Biochem J (1990) 265:621–36. 10.1042/bj2650621 1689567 PMC1133681

[5] Tenorio AR Zheng Y Bosch RJ Krishnan S Rodriguez B Hunt PW Soluble markers of inflammation and coagulation but not T-cell activation predict non-AIDS-defining morbid events during suppressive antiretroviral treatment. J Infect Dis (2014) 210:1248–59. 10.1093/infdis/jiu254 24795473 PMC4192039

[6] Boulware DR Hullsiek KH Puronen CE Rupert A Baker JV French MA Higher levels of CRP, D-dimer, IL-6, and hyaluronic acid before initiation of antiretroviral therapy (ART) are associated with increased risk of AIDS or death. J Infect Dis (2011) 203:1637–46. 10.1093/infdis/jir134 21592994 PMC3096784

[7] Brenchley JM Price DA Schacker TW Asher TE Silvestri G Rao S Microbial translocation is a cause of systemic immune activation in chronic HIV infection. Nat Med (2006) 12:1365–71. 10.1038/nm1511 17115046

[8] Jiang W Lederman MM Hunt P Sieg SF Haley K Rodriguez B Plasma levels of bacterial DNA correlate with immune activation and the magnitude of immune restoration in persons with antiretroviral-treated HIV infection. J Infect Dis (2009) 199:1177–85. 10.1086/597476 19265479 PMC2728622

[9] Siewe B Keshavarzian A French A Demarais P Landay A. A role for TLR signaling during B cell activation in antiretroviral-treated HIV individuals. AIDS Res Hum Retroviruses (2013) 29:1353–60. 10.1089/AID.2013.0115 23763346 PMC3785799

[10] Wilson EM Singh A Hullsiek KH Gibson D Henry WK Lichtenstein K Monocyte-activation phenotypes are associated with biomarkers of inflammation and coagulation in chronic HIV infection. J Infect Dis (2014) 210:1396–406. 10.1093/infdis/jiu275 24813472 PMC4207864

[11] Jalbert E Crawford TQ D’Antoni ML Keating SM Norris PJ Nakamoto BK IL-1beta enriched monocytes mount massive IL-6 responses to common inflammatory triggers among chronically HIV-1 infected adults on stable anti-retroviral therapy at risk for cardiovascular disease. PLoS One (2013) 8:e75500 10.1371/journal.pone.0075500 24086545 PMC3783392

[12] Anzinger JJ Butterfield TR Angelovich TA Crowe SM Palmer CS. Monocytes as regulators of inflammation and HIV-related comorbidities during cART. J Immunol Res (2014) 2014:569819. 10.1155/2014/569819 25025081 PMC4082935

[13] Kehrl JH Rieckmann P Kozlow E Fauci AS. Lymphokine production by B cells from normal and HIV-infected individuals. Ann N Y Acad Sci (1992) 651:220–7. 10.1111/j.1749-6632.1992.tb24617.x 1376041

[14] Barr TA Shen P Brown S Lampropoulou V Roch T Lawrie S B cell depletion therapy ameliorates autoimmune disease through ablation of IL-6-producing B cells. J Exp Med (2012) 209:1001–10. 10.1084/jem.20111675 22547654 PMC3348102

[15] Fillatreau S. Novel regulatory functions for toll-like receptor-activated B cells during intracellular bacterial infection. Immunol Rev (2011) 240:52–71. 10.1111/j.1600-065X.2010.00991.x 21349086

[16] Yeo L Lom H Juarez M Snow M Buckley CD Filer A Expression of FcRL4 defines a pro-inflammatory, RANKL-producing B cell subset in rheumatoid arthritis. Ann Rheum Dis (2014) 74:928–35. 10.1136/annrheumdis-2013-204116 24431391 PMC4392201

[17] Sohn HW Krueger PD Davis RS Pierce SK. FcRL4 acts as an adaptive to innate molecular switch dampening BCR signaling and enhancing TLR signaling. Blood (2011) 118:6332–41. 10.1182/blood-2011-05-353102 21908428 PMC3236118

[18] Kardava L Moir S Wang W Ho J Buckner CM Posada JG Attenuation of HIV-associated human B cell exhaustion by siRNA downregulation of inhibitory receptors. J Clin Invest (2011) 121:2614–24. 10.1172/JCI45685 21633172 PMC3127436

[19] Weiss GE Crompton PD Li S Walsh LA Moir S Traore B Atypical memory B cells are greatly expanded in individuals living in a malaria-endemic area. J Immunol (2009) 183:2176–82. 10.4049/jimmunol.0901297 19592645 PMC2713793

[20] Moir S Ho J Malaspina A Wang W DiPoto AC O’Shea MA Evidence for HIV-associated B cell exhaustion in a dysfunctional memory B cell compartment in HIV-infected viremic individuals. J Exp Med (2008) 205:1797–805. 10.1084/jem.20072683 18625747 PMC2525604

[21] Gowda DC. TLR-mediated cell signaling by malaria GPIs. Trends Parasitol (2007) 23:596–604. 10.1016/j.pt.2007.09.003 17980663

[22] Li FJ Schreeder DM Li R Wu J Davis RS. FCRL3 promotes TLR9-induced B-cell activation and suppresses plasma cell differentiation. Eur J Immunol (2013) 43:2980–92. 10.1002/eji.201243068 23857366 PMC3838486

[23] Ehrhardt GR Hijikata A Kitamura H Ohara O Wang JY Cooper MD. Discriminating gene expression profiles of memory B cell subpopulations. J Exp Med (2008) 205:1807–17. 10.1084/jem.20072682 18625746 PMC2525601

[24] Smolinska MJ Page TH Urbaniak AM Mutch BE Horwood NJ. Hck tyrosine kinase regulates TLR4-induced TNF and IL-6 production via AP-1. J Immunol (2011) 187:6043–51. 10.4049/jimmunol.1100967 22021612

[25] Kim MO Suh HS Si Q Terman BI Lee SC. Anti-CD45RO suppresses human immunodeficiency virus type 1 replication in microglia: role of Hck tyrosine kinase and implications for AIDS dementia. J Virol (2006) 80:62–72. 10.1128/JVI.80.1.62-72.2006 16352531 PMC1317521

[26] Fumagalli L Zhang H Baruzzi A Lowell CA Berton G. The Src family kinases Hck and Fgr regulate neutrophil responses to N-formyl-methionyl-leucyl-phenylalanine. J Immunol (2007) 178:3874–85. 10.4049/jimmunol.178.6.3874 17339487 PMC4683084

[27] Tak PP Firestein GS NF-kappaB: a key role in inflammatory diseases. J Clin Invest (2001) 107:7–11. 10.1172/JCI11830 11134171 PMC198552

[28] Firestein GS Manning AM Signal transduction and transcription factors in rheumatic disease. Arthritis Rheum (1999) 42:609–21. 10.1002/1529-0131(199904)42:4<609::AID-ANR3>3.0.CO;2-I 10211874

[29] Kim JH Song AR Sohn HJ Lee J Yoo JK Kwon D IL-1beta and IL-6 activate inflammatory responses of astrocytes against *Naegleria fowleri* infection via the modulation of MAPKs and AP-1. Parasite Immunol (2013) 35:120–8. 10.1111/pim.12021 23198898

[30] Amu S Lavy-Shahaf G Cagigi A Hejdeman B Nozza S Lopalco L Frequency and phenotype of B cell subpopulations in young and aged HIV-1 infected patients receiving ART. Retrovirology (2014) 11:76 10.1186/s12977-014-0076-x 25213015 PMC4172851

[31] Jelicic K Cimbro R Nawaz F Huang da W Zheng X Yang J The HIV-1 envelope protein gp120 impairs B cell proliferation by inducing TGF-beta1 production and FcRL4 expression. Nat Immunol (2013) 14:1256–65. 10.1038/ni.2746 24162774 PMC3870659

[32] Won WJ Foote JB Odom MR Pan J Kearney JF Davis RS. Fc receptor homolog 3 is a novel immunoregulatory marker of marginal zone and B1 B cells. J Immunol (2006) 177:6815–23. 10.4049/jimmunol.177.10.6815 17082595

[33] Liu Y Bezverbnaya K Zhao T Parsons MJ Shi M Treanor B Involvement of the HCK and FGR src-family kinases in FCRL4-mediated immune regulation. J Immunol (2015) 194:5851–60. 10.4049/jimmunol.1401533 25972488 PMC4456631

