# Supplementary figures and images for: FcRL4 Expression Identifies a Pro-inflammatory B Cell Subset in Viremic HIV-Infected Subjects
Source: Front Immunol. 2017 Oct 20;8:1339. doi: 10.3389/fimmu.2017.01339 (PMC5655023; doi:10.3389/fimmu.2017.01339)

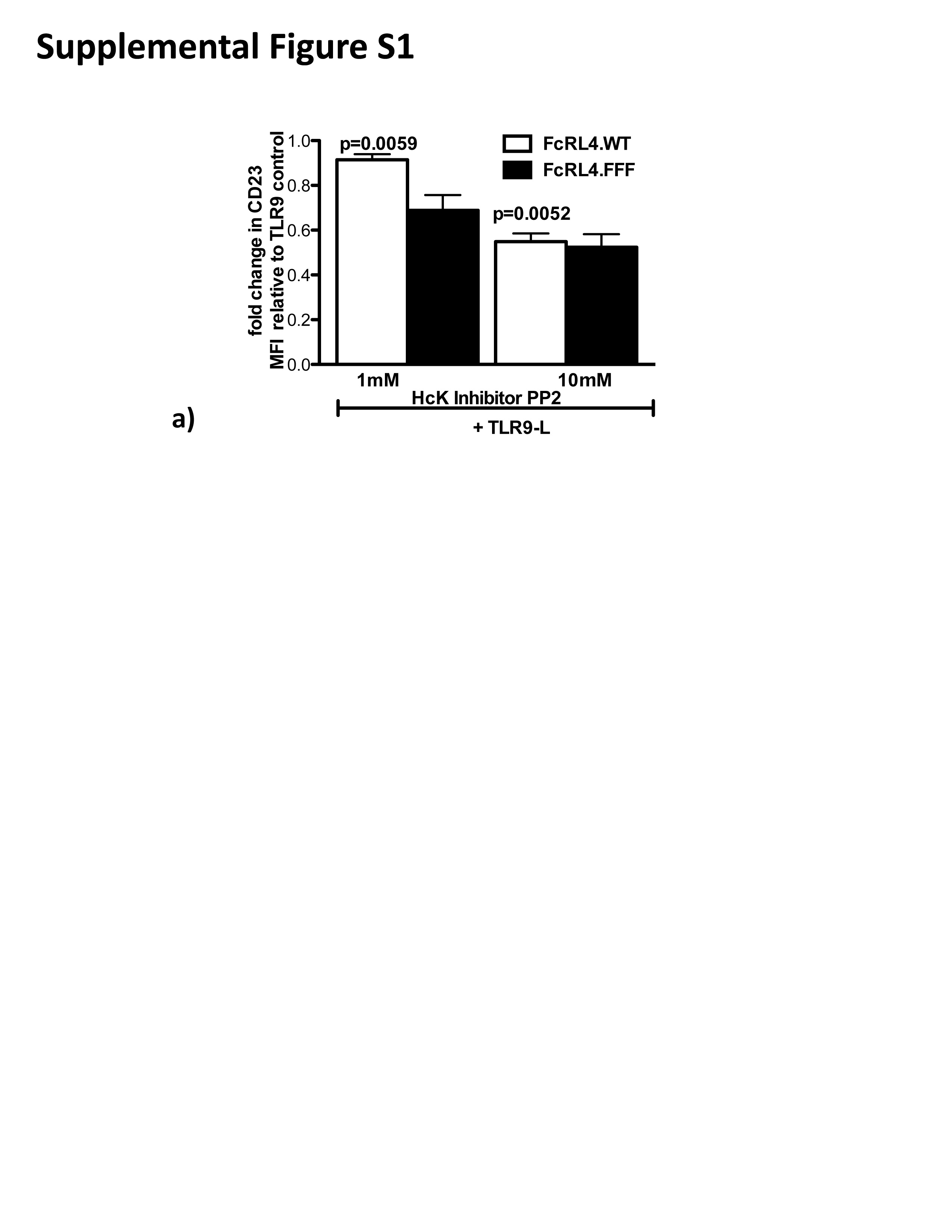

Supplement: Figure S1 — CD23 expression following overnight treatment with HcK inhibitor PP2. FcRL4.WT and FcRL4.FFF cells were treated with TLR9-L and HcK inhibitor. Expression of CD23 reported as fold change between TLR9-L alone and in the presence of inhibitor n=3. [file image_1.jpeg]

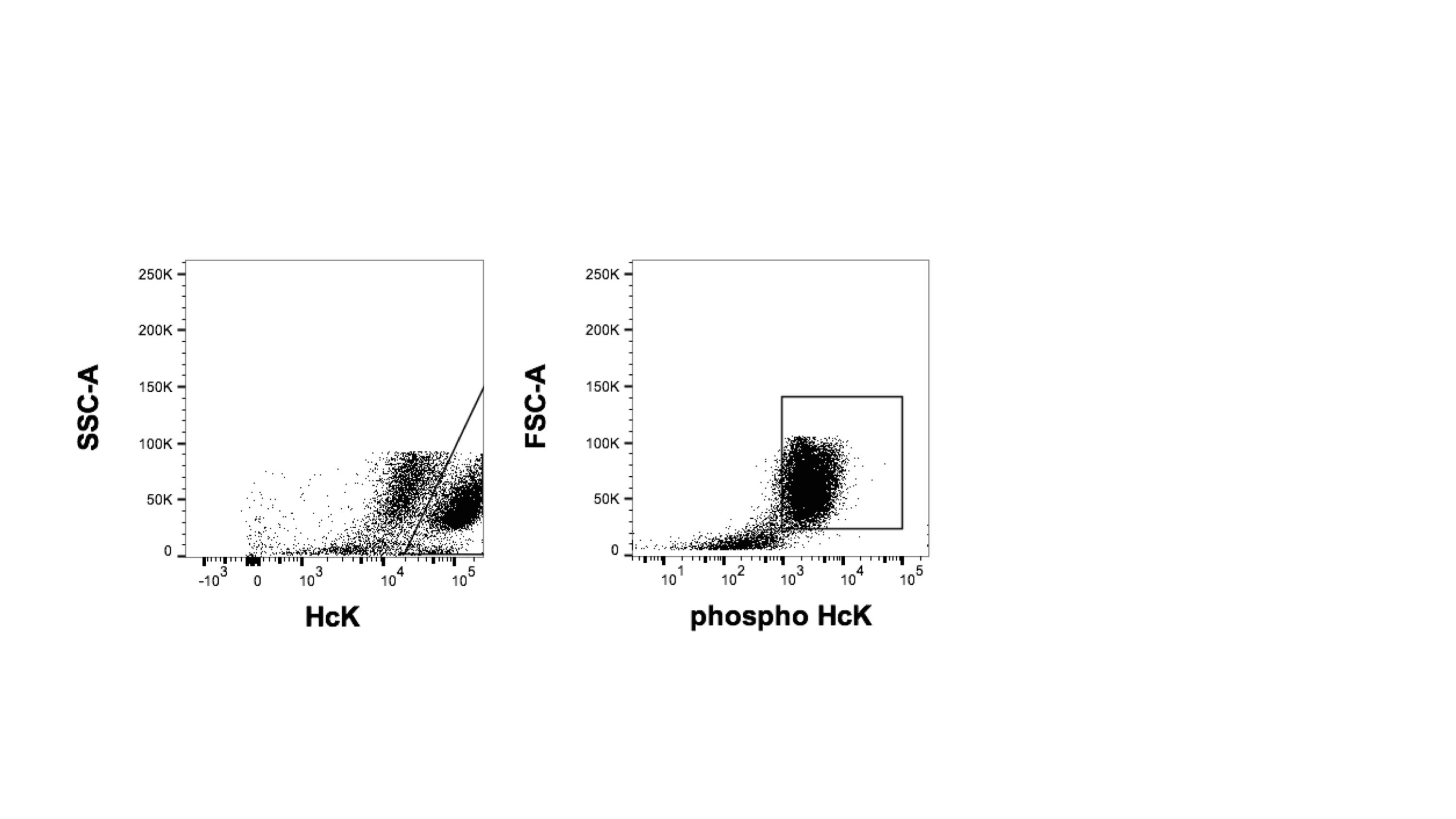

Supplement: Figure S2 — Representative gating of HcK and phospho HcK. [file image_2.jpeg]
